# Immune Dysfunction and Coinfection with Human Immunodeficiency Virus and *Schistosoma japonicum* in Yi People

**DOI:** 10.1155/2018/6989717

**Published:** 2018-07-02

**Authors:** Yu Yang, Peng-Lei Xiao, Ya Yang, Jian-Chuan Gao, Yan Shi, Wan-Ting Cheng, Yue Chen, Xiu-Xia Song, Qing-Wu Jiang, Yi-Biao Zhou

**Affiliations:** ^1^Department of Epidemiology, School of Public Health, Fudan University, 138 Yi Xue Yuan Road, Shanghai 200032, China; ^2^Key Laboratory of Public Health Safety, Fudan University, Ministry of Education, Shanghai, China; ^3^Center for Tropical Disease Research, Fudan University, Shanghai, China; ^4^School of Epidemiology and Public Health, Faculty of Medicine, University of Ottawa, Ottawa, ON, Canada

## Abstract

**Objective:**

To explore the association between infections with HIV and *Schistosoma japonicum*, and to determine the influences of the HIV-*S. japonicum* coinfections on the immune system of Yi people.

**Methods:**

A block design study was conducted in a Yi county in southwestern China, one of the endemic areas of both HIV/AIDS and *S. japonicum* in China. All participants were screened for HIV antibodies and *S. japonicum* antibodies (SjAb) and were classified into four groups: HIV(+)/*S. japonicum*(−), HIV(−)/*S. japonicum* (+), HIV(+)/*S. japonicum*(+), and HIV(−)/*S. japonicum*(−).

**Results:**

There were significant differences among the four groups in both CD4^+^ T lymphocytes and CD8^+^ T lymphocytes, but no significant difference in CD3^+^ T lymphocytes. Both the CD4^+^ T lymphocyte counts and the ratio of CD4^+^/CD8^+^ were lower in HIV-infected people compared with those uninfected. People infected with *S. japonicum* had increased CD4^+^ T lymphocyte counts but reduced CD8^+^ T lymphocyte counts. Similarly, the ratio of CD4^+^/CD8^+^ was higher in *S. japonicum*-infected people compared with those uninfected. People coinfected with HIV and *S. japonicum* had lower CD4^+^ T lymphocyte counts, lower ratio of CD4^+^/CD8^+^, and higher CD8^+^ T lymphocyte counts compared with those infected with HIV only or *S. japonicum* only. People infected with HIV only and those coinfected with HIV and *S. japonicum* had a higher level of IFN-*γ* compared with people with no infection. There were no significant differences between people infected with HIV only and with *S. japonicum* only in the levels of IFN-*γ* and IL-10.

**Conclusions:**

People coinfected with HIV and *S. japonicum* might have a suppressed immune function because of a decrease in CD4^+^ T lymphocyte counts, a lowered ratio of CD4^+^/CD8^+^, and an increase in CD8^+^ T lymphocyte counts. Coinfection with HIV and *S. japonicum* would alter the level of IFN-*γ* in plasma.

## 1. Introduction

Human immunodeficiency virus (HIV) is a global health problem, as are helminth infections. Schistosomiasis is one of the chronic, water-borne helminth diseases [1], and it is a risk factor for HIV infection [2]. People infected with *S. mansoni* or *S. haematobium* are more susceptible to HIV infection due to common high-risk behaviors, such as having multiple sexual partners and other exposures to sexually transmitted diseases [3–5]. There is also an overlap of multiple risk factors associated with the HIV and *S. mansoni* infections in the same geographical setting or the biological interaction between them to increase the risk of individuals to be coinfected with both [3, 4, 6–8]. Epidemiological studies have reported that there is an association between HIV infection and schistosomiasis [8–11].

In early 1990, some researchers found that animals infected with *Schistosoma mansoni* could produce antibodies that was specific to one protein of HIV, the regulatory protein virion infectivity factor (VIF), and the VIF could identify a 170 kDa peptide of *S. mansoni* [12]. In humans, a study conducted in rural Tanzanian villages near Lake Victoria found that *S. mansoni* infection predicted HIV infection among reproductive age women [13]. Besides, urogenital schistosomiasis may be a risk factor for HIV infection [14, 15]. Furthermore, some studies have reported that HIV increases the risk of parasite infection as HIV attacks the human immune system and causes cellular immunity dysfunction [16]. People infected with parasites are also at higher risk for HIV infection compared with those uninfected [16, 17]. Infections of schistosomiasis and with HIV can be mutually promoted through the immunological interactions [18, 19]. Cytokines play an important role in both antiviral and antiparasitic diseases. The HIV- (nonenvelope) specific antiviral T-cell immune response is dominated by the secretion of IFN-*γ*, TNF-*α* (Th1 profile) [4], and IL-17 (Th17 profile), whereas *S. mansoni* infection in humans is predominantly characterized by the secretion of IL-4, IL-5, IL-13 (Th2 profile), and IL-17 (Th17 profile) in the acute phase and a regulatory phenotype (T regs) in the chronic phase [20]. IL-17 is also a critical mediator of liver fibrosis in *S. japonicum*-infected mice [21]. Both HIV and schistosome infection cause increased levels of IFN-*γ* and IL-10 [22–25]. After HIV and schistosome infection, the balance of the immune state is maintained by upregulating the expression of IFN-*γ* [26, 27].

CD4^+^ T cells expressing the chemokine receptor CCR5 are the predominant targets of HIV during initial infection, and specific CD4^+^ T helper (Th) subsets are particularly susceptible to HIV [28–30]. *In vitro* studies demonstrated that patients with active schistosomiasis displayed higher cell surface densities of chemokine receptors CCR5 and CXCR4, making the cells more susceptible to HIV than those from helminth-free individuals [31]. As HIV infection is associated with reduced CD4^+^ T lymphocyte counts, it was previously reported that the destruction of helper CD4^+^ lymphocytes by the HIV virus in coinfected individuals could affect granuloma formation of *S. mansoni* infection and alter the egg excretion efficiency [10, 32]. Granuloma formation in *S. mansoni* infection is a CD4^+^ T lymphocyte-dependent process [32]. Some earlier studies have hypothesized that the destruction of helper CD4^+^ T lymphocytes (Th2) by HIV, coupled with the significant importance of CD4^+^ cells in the formation of granuloma, may lead to a decreased ability of the Th2 aiming to produce proinflammatory cytokines, and hence lead to severe hepatic morbidity [33, 34]. HIV-infected patients with *S. mansoni* coinfection also displayed a significantly higher number of Gag-specific IL-10-positive CD8^+^ T cells. Immunological studies have also found the biological mechanisms through which chronic HIV infection could affect *S. mansoni*-related morbidities [12, 31]. These mechanisms could result in differences in the prevalence and intensity of *S. mansoni* infection, the efficiency of parasite egg excretion, morbidity patterns, and the response to anthelmintic treatment among HIV-infected and noninfected people [9, 31, 35]. Similar observations on the influence of egg excretion were reported in HIV-1-positive individuals coinfected with *S. mansoni* or *S. haematobium* in Ethiopia, Kenya, and Uganda [9, 36, 37]. However, some studies found that *S. mansoni* infection was not associated with HIV acquisition. *S. haematobium* causes urogenital schistosomiasis and poses a risk for HIV acquisition through the urogenital lesions [15, 38]. As opposed to *S. haematobium*, *S. mansoni* is unlikely to cause genital lesions to have an impact on HIV-1 acquisition [39]. Other studies suggested that systemic immune modulation by *S. mansoni* might not significantly increase the susceptibility to HIV acquisition [9, 39].

To our knowledge, the impact of *S. japonicum* and HIV coinfection on immune responses has not been fully assessed. Hence, in this study, we investigate the possible association between the infections with HIV and *S. japonicum* by exploring the alteration of CD4^+^ and CD8^+^ T lymphocyte counts and cytokine levels in coinfected individuals compared with those uninfected or infected with HIV or *S. japonicum* alone.

## 2. Materials and Methods

### 2.1. Study Field

This study was conducted in Puge County of the Liangshan Yi Prefecture, southwestern China, from July 22nd to August 11th, 2015. It is an underdeveloped region inhabited mainly by the Yi people, an ethnic minority group in China. In this mountainous region of southwestern China, the high risk of HIV infection is mainly due to drug abuse and the casual sexual behavior of the “Yi people” [40]. This “Yi” county has a complex topography with mountains and valleys with an average elevation of 1800 meters. The local climate is subtropical monsoon with mild winters and warm and humid summers. These special geographical and environmental conditions are suitable for *S. japonicum* to grow and spread. Infections with *S. japonicum* are also common due to poor sanitation conditions and weak health infrastructure.

### 2.2. Participants

A total of 90 Yi individuals was recruited from the county above. Participants had to be Yi people aged >3 years without metabolic or autoimmune disease. Pregnant women and those with severe organic or mental diseases or any known medical problem were excluded. Individuals who did not have T lymphocyte or cytokine test or *Schistosoma* antibody test results were also excluded.

### 2.3. Study Procedures

**Figure 1:**
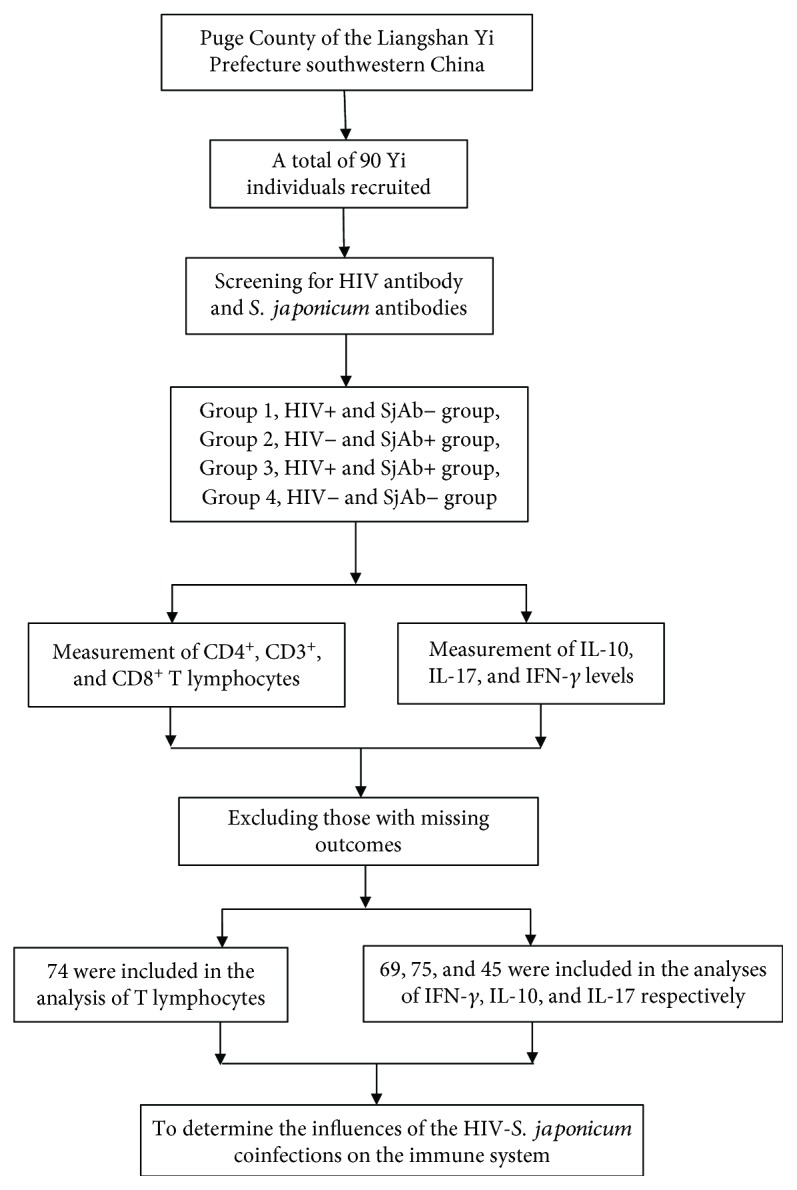
The research process.

Figure 1 showed the research process. Health workers who participated in this investigation were trained under the guidance of a unified protocol. They informed all the potential participants and explained the objectives, procedures, and potential risks of the study. Written consent was obtained from all adult participants and from the parents or legal guardians of children. A data collection sheet covered information on sociodemographic factors, details of HIV and *S. japonicum* infections, and other conditions.

All participants were asked to provide a 5 ml blood specimen for measurements of CD4^+^ and CD8^+^ T lymphocyte counts and cytokine levels like IL-10, IL-17, and IFN-*γ*. T lymphocyte counts were enumerated in EDTA blood by using Becton Dickinson (BD) FACScount (version 1.5; BD FACScount™ controls, catalogue number: 340166; BD FACScount reagents, catalogue number: 340167). Plasma was stored at −80°C for cytokine quantification, which was performed by the use of a human Multiplex Immunoassay (ProcartaPlex Mix&Match Human 5-plex, eBioscience Inc., Vienna, Austria (with the headquarters at San Diego, CA), catalogue number: EPX050-15122-801). A total of 50 *μ*l of plasma sample was used to quantify the related cytokines in the samples.

All the participants were screened for HIV antibody by using the Diagnostic Kit for Antibody to HIV (colloidal gold) (a product of InTec Products Inc., Xiamen, P. R. China, batch number: 20150420, 100 persons per kit). Those positive of HIV antibody were further tested with HIV RNA by using the diagnostic kit for the quantification of HIV RNA (PCR Fluorescence Probing) (a product of the Da An Gene Group Inc., Zhongshan, P. R. China, batch number: 2015002, 48 persons per kit). Participants were also screened for *S. japonicum* antibodies (SjAb) by using the Indirect Hemagglutination Assay (IHA) kit (a product of the Anji Pharmaceutical Group Co., Ltd, Anhui, P. R. China, batch number: 20150200, 100 persons per kit). Those positive for SjAb were asked to provide a faecal sample of at least 30 g collected in the morning at home, and an oral description and specific instructions for handling and contamination avoidance of the stool sample were given. All the samples were sent to the laboratory of the local CDC for examination as soon as possible after they were collected. The faecal samples were processed within 12 h postcollection by using the stool hatching method for the detection of *S. japonicum* miracidia. Every sample was initially read by two examiners and reviewed by a third examiner if there was a disagreement.

### 2.4. Statistical Analysis

Data were double-entered and cross-checked by using the EpiData software (version 3.1; The EpiData Association, Odense, Denmark). Kruskal-Wallis rank tests were used to compare median CD4^+^ and CD8^+^ T lymphocyte counts and the levels of cytokines IL-10, IL-17, and IFN-*γ*. A two-sided *P* value of 0.05 or less was regarded as significant. Statistical analyses were carried out with SPSS (version 20.0; IBM SPSS Institute Inc., USA).

## 3. Results

A total of 90 participants were recruited and they were classified into four groups: HIV+/SjAb− (*n* = 32), HIV−/SjAb+ (*n* = 13), HIV+/SjAb+ (*n* = 16), and HIV−/SjAb− (*n* = 29). After excluding those with missing outcomes, 74 were included in the analysis of T lymphocytes (Table 1) and 69, 75, and 45 were included in the analyses of IFN-*γ*, IL-10, and IL-17, respectively (Table 2). All of the SjAb-positive participants were examined for *S. japonicum* miracidia by a stool-hatching method, and no active miracidia were found.

**Figure 2:**
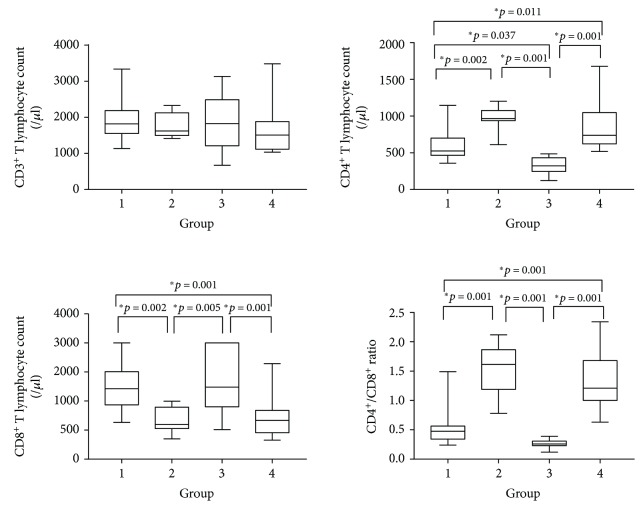
Comparison of T lymphocytes among 4 groups. Data are presented as the min to max.

The median of the participants was 34.5 years and interquartile range (IQR) was 24.7–41.2. There were no significant differences among the four groups in age, gender, occupation, and education (Tables 1 and 2).  Figure 2 showed that there were significant differences among the four groups in both CD4 and CD8^+^ T lymphocytes but no significant differences in CD3^+^ T lymphocytes. These results suggested that infection with HIV might reduce the CD4^+^ T lymphocyte counts but increase the counts of CD8^+^ T lymphocytes. The ratio of CD4/CD8 was lower in HIV-infected people compared with those who were uninfected. Infection with *S. japonicum* was associated with increased CD4^+^ T lymphocyte counts but with reduced CD8^+^ T lymphocyte counts. Similarly, the ratio of CD4^+^/CD8^+^ was higher in people infected with *S. japonicum* compared with those uninfected. Coinfection with HIV and *S. japonicum*, similar to HIV infection alone, was significantly associated with lymphocyte counts. People with coinfection had lower CD4^+^ T lymphocyte counts, a lower ratio of CD4/CD8, and higher CD8^+^ T lymphocyte counts compared with those infected with HIV only or *S. japonicum* only.

**Figure 3:**
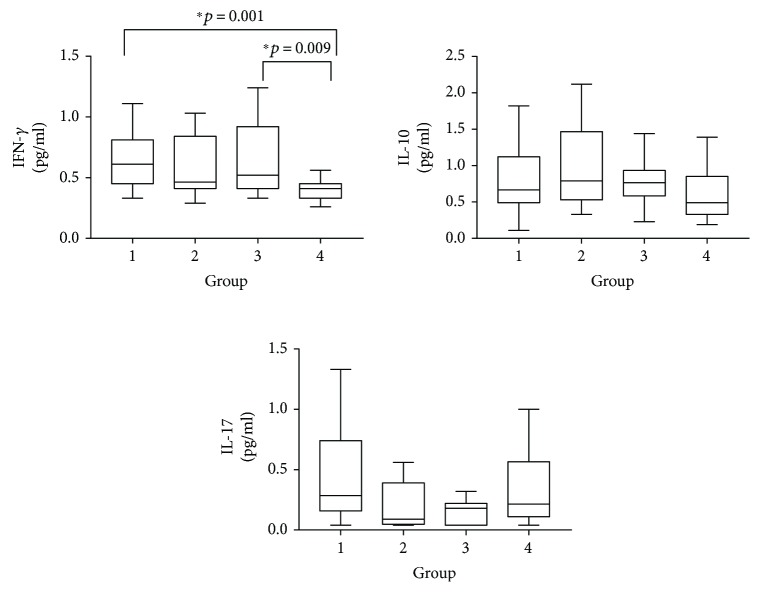
Comparison of IFN-*γ*, IL-10, and IL-17 levels among 4 groups. Data are presented as the min to max.

Figure 3 presents the levels of IFN-*γ*, IL-10, and IL-17 and comparisons among the four groups. There was no significant difference in IL-17 among the four groups (*Z* = 5.255, *P* = 0.154), while there were significant differences among the four groups for both IFN-*γ* and IL-10. People infected with HIV and those who were coinfected with HIV and *S. japonicum* had a higher level of IFN-*γ* compared with people with no infection. There were no significant differences between people infected with HIV only and with *S. japonicum* only in the levels of IFN-*γ* and IL-10.

## 4. Discussion

Studies have suggested that HIV infection damages the immune system and decreases CD4^+^ T lymphocyte counts of the human body [16]. We found that people infected with HIV had higher CD8^+^ T lymphocyte counts compared with those who were uninfected. In our study, participants were relatively young (33.5 (24.00, 39.25) years) with a short average infection duration of HIV (42.65 ± 21.52 months). Consistent with previous findings, HIV-infected people had higher levels of CD8^+^ T lymphocytes compared with uninfected people [41]. In the early stage of HIV infection, the immune system may not be seriously damaged and may be in the condition of stress response state. During such phase, uncontrolled replication of HIV-1 infection leads to activations of the CD8^+^ T lymphocytes (which can inhibit HIV replication by cytolytic and noncytolytic responses) and increases the concentration of cytokines such as IFN-*γ* and IL-10 [42, 43]. Earlier studies demonstrated a correlation of maintaining the CD8^+^ T lymphocyte immune profile with a slow progression of HIV-1 infection [44, 45].

CD4^+^ T lymphocytes also play an important role in the human body for the balance of immune responses, and is a widely used marker of HIV immune impairment [46]. We found that people who were coinfected with HIV and *S. japonicum* had a lower level of CD4^+^ T lymphocyte counts and a higher level of CD8^+^ T lymphocyte counts. Coinfection with HIV and *S. japonicum* might speed up the disease progression of HIV infection. Destruction of the CD4^+^ T cell pool increases susceptibility of the host to other infectious diseases [17].

We did not find that infection with *S. japonicum* exerted a significant impact on the level of T lymphocytes. People infected with *S. japonicum* had a slightly higher level of CD4^+^ T lymphocyte counts and a lower level of CD8^+^ T lymphocyte counts compared with people with no infection, but these were not statistically significant. The CD4^+^ T lymphocyte responses are central to the development of immunopathology in *S. mansoni* or *S. japonicum* infections [47, 48]. At a chronic phase of infections, the Th1 response will shift to a Th2 response (characterized by the upregulation of IL-10 and other cytokines), which downregulates the production and function of the Th1 response [49], accompanied by a dysfunction of cellular immunity, like the apoptosis of CD4^+^ T lymphocytes and activation of CD8^+^ T lymphocytes [50, 51]. A possible explanation of this inconsistency might be that the SjAb+ subjects in this research were all asymptomatic and had no active miracidia. Therefore, they might be in an early stage of infection or were previously infected. At the early stage of schistosome infections, immunity dysfunction might not occur and the level of T lymphocytes did not change substantially. A standard treatment of schistosome infection could switch the immune system back to normal. It was reported that Th1 type responses, which are characterized by producing IFN-*γ*, were predominated by other than Th2 type responses in the early stage of schistosome infections [52]. Consistently, we found that SjAb+ individuals had a higher level of IFN-*γ*. Since Th1 cells primarily mediate cellular immunity, arousing the increase of CD4^+^ T lymphocytes while CD8^+^ T lymphocytes are not yet activated or just in the stress state [51, 53], no significant changes in the levels of CD4^+^ T lymphocytes and CD8^+^ T lymphocytes were shown in the current study.

In our study, people infected with HIV only had a higher level of IFN-*γ* compared with people with no infection with HIV and *S. japonicum*. Individuals coinfected with both HIV and *S. japonicum* had similar results when compared with people with no infection with HIV and *S. japonicum*; however, they had no higher levels of IFN-*γ* or IL-10 compared with individuals infected with HIV or *S. japonicum* alone. A possible explanation for this was that people might be in the early stage of infection with *S. japonicum* or taking praziquantel for the treatment of schistosomiasis that could impact the levels of IL-10 and IFN-*γ* in plasma [10]. We found no significant difference in the level of IL-17 among the four groups.

The small sample size is a main limitation for the study, largely because of the challenging conditions in the study field (i.e., due to the effective measurements for the prevention and control *Schistosoma japonicum*, it is rather difficult to find enough cases to conduct this research), and we were not able to explore the interactions between the infections in different stages. We could not control for potential impacts from other pathogens on the results. The levels of three cytokines in the study were lower compared with those previously reported.

## 5. Conclusions

People coinfected with HIV and *S. japonicum* might have a suppressed immune function because of a decrease in CD4^+^ T lymphocyte counts, a lowered ratio of CD4^+^/CD8^+^, and an increase in CD8^+^ T lymphocyte counts. Coinfection with both HIV and *S. japonicum* would alter the level of IFN-*γ* in plasma. No difference in the level of IL-17 was detected among the four study groups.

## Figures and Tables

**Table 1:** Characteristics of individuals included in the analysis of T lymphocytes.

Characteristic		Group 1 (*n* = 24)	Group 2 (*n* = 12)	Group 3 (*n* = 11)	Group 4 (*n* = 27)	*z*/*χ*^2^	*P*
Age, median (interquartile range), years		33.5 (24.00, 39.25)	36.00 (15.25, 44.75)	36.00 (33.00, 40.00)	35.00 (18.00, 45.00)	1.920^∗^	0.559
Sex	Male	17	9	5	15		0.348^∗∗^
	Female	7	3	6	12		
Education	Illiterate	12	5	8	9		0.208
	Elementary school and above	12	7	3	17		
Occupation	Peasant	21	9	10	18		0.341^∗∗^
	Else	3	3	1	8		

*Note*. Group 1, the HIV+ and SjAb− group; Group 2, the HIV− and SjAb+ group; Group 3, the HIV+ and SjAb+ group; Group 4, the HIV− and SjAb− group; ^∗^Kruskal-Wallis rank test, ^∗∗^Fisher's exact test.

**Table 2:** Characteristics of individuals included in the analysis of IFN-*γ*, IL-10, and IL-17 levels.

IFN-*γ*							
Characteristic		Group 1 (*n* = 19)	Group 2 (*n* = 12)	Group 3 (*n* = 15)	Group 4 (*n* = 23)	*z*/*χ*^2^	*P*
Age, median (interquartile range), years		34 (8.00, 40.00)	36.00 (16.00, 44.75)	36.00 (33.00, 40.00)	36.00 (23.00, 45.00)	2.161^∗^	0.540
Sex	Male	12	10	8	11		0.216^∗∗^
	Female	7	2	7	12		
Education	Illiterate	10	4	9	7	4.009	0.260
	Elementary school and above	9	8	6	15		
Occupation	Peasant	16	9	13	17		0.843^∗∗^
	Else	3	3	2	5		

IL-10							
Characteristic		Group 1 (*n*=22)	Group 2 (*n*=12)	Group 3 (*n*=14)	Group 4 (*n*=27)	*z*/*χ*^2^	*P*
Age, median (interquartile range), years		34.00 (19.25, 40.50)	36.00 (16.00, 44.75)	36.00 (32.75, 40.25)	35.00 (18.00, 45.00)	1.074^∗^	0.783
Sex	Male	15	10	9	15		0.419^∗∗^
	Female	7	2	5	12		
Education	Illiterate	11	4	9	9	4.460	0.216
	Elementary school and above	11	8	5	18		
Occupation	Peasant	18	9	12	19		0.776^∗∗^
	Else	4	3	2	8		

IL-17							
Characteristic		Group 1 (*n* = 16)	Group 2 (*n* = 8)	Group 3 (*n* = 7)	Group 4 (*n* = 14)	*z*/*χ*^2^	*P*
Age, median (interquartile range), years		32.00 (7.25, 41.50)	29.50 (9.75, 44.75)	35.00 (30.75, 38.00)	25.50 (7.75, 34.50)	3.612^∗^	0.307
Sex	Male	11	7	4	8		0.363^∗∗^
	Female	5	1	3	6		
Education	Illiterate	9	2	5	4		0.324^∗∗^
	Elementary school and above	7	6	2	9		
Occupation	Peasant	12	5	6	9		0.869^∗∗^
	Else	4	3	1	4		

^∗^Kruskal-Wallis rank test, ^∗∗^Fisher's exact test.

## Data Availability

The data used to support the findings of this study are available from the corresponding author upon request.

## Abbreviations

HIV: Human immunodeficiency virus
AIDS: Acquired immunodeficiency symptom
SjAb: *Schistosoma japonicum* antibody
IHA: Indirect hemagglutination assay
RNA: Ribonucleic acid
CD: Cluster of differentiation
Th: T helper lymphocyte
IFN: Interferon
IL: Interleukin
CI: Confidence interval
OR: Odds ratio.
